# Recruitment Kinetics of DNA Repair Proteins Mdc1 and Rad52 but Not 53BP1 Depend on Damage Complexity

**DOI:** 10.1371/journal.pone.0041943

**Published:** 2012-07-30

**Authors:** Volker Hable, Guido A. Drexler, Tino Brüning, Christian Burgdorf, Christoph Greubel, Anja Derer, Judith Seel, Hilmar Strickfaden, Thomas Cremer, Anna A. Friedl, Günther Dollinger

**Affiliations:** 1 Angewandte Physik und Messtechnik LRT2, UniBw-München, Neubiberg, Germany; 2 Klinik und Poliklinik für Strahlentherapie und Radioonkologie, LMU-München, München, Germany; 3 Department Biologie II, LMU-München, Martinsried, Germany; University of Medicine and Dentistry of New Jersey, United States of America

## Abstract

The recruitment kinetics of double-strand break (DSB) signaling and repair proteins Mdc1, 53BP1 and Rad52 into radiation-induced foci was studied by live-cell fluorescence microscopy after ion microirradiation. To investigate the influence of damage density and complexity on recruitment kinetics, which cannot be done by UV laser irradiation used in former studies, we utilized 43 MeV carbon ions with high linear energy transfer per ion (LET = 370 keV/µm) to create a large fraction of clustered DSBs, thus forming complex DNA damage, and 20 MeV protons with low LET (LET  = 2.6 keV/µm) to create mainly isolated DSBs. Kinetics for all three proteins was characterized by a time lag period T_0_ after irradiation, during which no foci are formed. Subsequently, the proteins accumulate into foci with characteristic mean recruitment times τ_1_. Mdc1 accumulates faster (T_0_ = 17±2 s, τ_1_ = 98±11 s) than 53BP1 (T_0_ = 77±7 s, τ_1_ = 310±60 s) after high LET irradiation. However, recruitment of Mdc1 slows down (T_0_ = 73±16 s, τ_1_ = 1050±270 s) after low LET irradiation. The recruitment kinetics of Rad52 is slower than that of Mdc1, but exhibits the same dependence on LET. In contrast, the mean recruitment time τ_1_ of 53BP1 remains almost constant when varying LET. Comparison to literature data on Mdc1 recruitment after UV laser irradiation shows that this rather resembles recruitment after high than low LET ionizing radiation. So this work shows that damage quality has a large influence on repair processes and has to be considered when comparing different studies.

## Introduction

Various proteins are involved in the cellular reactions to double-strand breaks (DSBs) induced by ionizing radiation [1]. Their functions range from signalling to DSB repair. Many of these proteins accumulate in the vicinity of the break site, forming so-called radiation-induced foci that can be visualized by immunofluorescence or live-cell imaging methods [2], [3]. Often there are complex dependencies between the various proteins, and the analysis of the kinetics of recruitment has in the past helped in understanding these mutual dependencies [2]. By using live-cell imaging methods, the subcellular localization and dynamics of foci-forming proteins can be studied in real time, starting immediately after damage infliction. In combination with localized damage induction where time and localization of the damage are pre-determined, very accurate measurements are possible [4]. In the last years, a detailed picture of the protein migrations and post-translational modifications occurring within the first seconds to minutes after DSB induction has emerged [2], [5] which is largely based on data obtained after localized irradiation with laser microbeams. A variety of laser microirradiation set-ups have been described to induce, in addition to other DNA damage types, DSBs [6], [7]. A disadvantage of the laser-based methods depends on the difficulty to predict the amount and distribution of damage types induced by a certain set-up and to compare the results obtained with different set-ups. Some attempts were made to calibrate laser-induced damage by comparison with damage induced by ionizing radiation, e.g. by comparing DNA fragmentation [7] or foci induction [8]. Irradiation with ionizing radiation has the advantage that the dose deposited at the irradiated region can accurately be determined and that detailed knowledge on amount and types of DNA lesions thus induced is available. In addition, ionizing radiation is a relevant genotoxic agent to which everybody is exposed in everyday life, e.g. through natural background radiation or medical applications. So far, however, only few facilities have been described that combine ionizing microirradiation with online live-cell microscopy [9], [10], [11], [12], [13]. At the Munich ion microbeam facility SNAKE (superconducting nanoprobe for applied nuclear (German: kern-) physics experiments), microirradiation of cells at submicrometer resolution [14] is combined with online fluorescence microscopy [10] to allow for the analysis of protein recruitment at damage sites induced by transversal of ions. A broad spectrum of ions is available, from 4–25 MeV protons to 40–200 MeV heavy ions, which makes it possible to investigate whether radiation quality affects the kinetics of recruitment of damage response proteins. Indications for such a dependence on radiation quality had been suggested in the past [3], but a detailed comparison study has so far not been presented.

The complexity of DNA damage is expected to be much larger for high linear energy transfer (LET) irradiation than for low LET irradiation. Of special relevance are clustered DSB, defined here as the occurrence of >1 DSB within a chromatin region corresponding to a radiation-induced focus. It was repeatedly observed that the number of foci along ion tracks falls short of the number of DSB expected to occur in the track, strongly suggesting the presence of several DSB within one focus [15], [16], [17]. Monte Carlo simulations show, as an example, that the average number N_cluster_ of DSBs within one DSB cluster defined as all DSBs along one 150 nm long fiber section (corresponding to 1.8 × 10^4^ base pairs) amounts to N_cluster_  = 2.2 for 75 MeV carbon ions at LET  = 250 keV/µm, while individual DSBs are nearly isolated (N_cluster_ <1.1 DSBs) on such a section when irradiating with 20 MeV protons at LET  = 2.6 keV/µm [18]. In the present work, we use 43 MeV carbon ions and 20 MeV protons to investigate recruitment kinetics in dependence of LET and thus complexity of DNA damage.

We study the recruitment of three proteins known to exhibit different recruitment kinetics: Mdc1, 53BP1, and Rad52. Mdc1 is a large mediator/adaptor protein playing a key role in the assembly of radiation-induced foci [5] and it is one of the earliest factors found to accumulate at DSB sites [19], [20], [21], [22]. Persistent presence of Mdc1 in foci depends on the presence of γ-H2AX, i.e. histone H2AX phosphorylated at Serine 139 [22]. Mdc1 binds directly to the C-terminal end of γ-H2AX containing the phosphorylated serine [23], [24].The recruitment of the mediator protein 53BP1, while still not mechanistically elucidated in every detail (for recent reviews see [2], [25]), appears to depend on a cascade of prior protein recruitment, modification, and removal steps, which explains why 53BP1 foci were consistently found to arise with a certain delay as compared to Mdc1 foci [26], [27]. Rad52, a protein involved in DSB repair by homologous recombination, has been reported to form visible foci with an extended delay in the range of hours [28], [29]. Here, we show that the mean recruitment time depends on LET and dose in the case of Mdc1 and Rad52, but not in the case of 53BP1. For all three proteins, however, the initial delay phase is influenced by LET.

## Results

### Analyzing and Modeling Protein Kinetics

**Figure 1:**
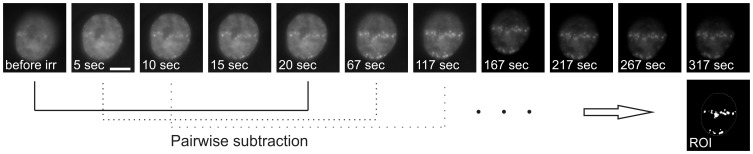
Parts of a micrograph time series of a U2OS cell nucleus showing GFP-tagged protein Mdc1. Irradiation took place on t  = 0. Foci formation can be observed already a few seconds after irradiation. Pairwise subtraction of the images reveals areas where foci are formed. The merge of these areas results in the region of interest (ROI) in which the foci brightness I_foci_ is evaluated for each image of the time series (cf. materials and methods). Scale bar in second image: 5 µm.

U2OS or HeLa cells with GFP tagged repair proteins Mdc1, 53BP1 and Rad52 were irradiated at the ion microprobe SNAKE with 43 MeV carbon ions respectively 20 MeV protons in a line shaped pattern. By varying the geometry of the pattern or the number of protons applied to one point of the pattern the dose can be adjusted. Immediately after irradiation microscopic time series of the irradiated cells were taken (fig. 1). From these images the kinetics is evaluated by measuring the mean intensity I_foci_(t) per pixel of the foci sites (i.e. the region of interest ROI in fig. 1, see also Materials and Methods). To correct for photobleaching this value is normalized to the mean intensity per pixel I_nucl_(t) of the whole cell nucleus for each image, resulting in I_rel_(t)  =  I_foci_(t)/I_nucl_(t).

**Figure 2:**
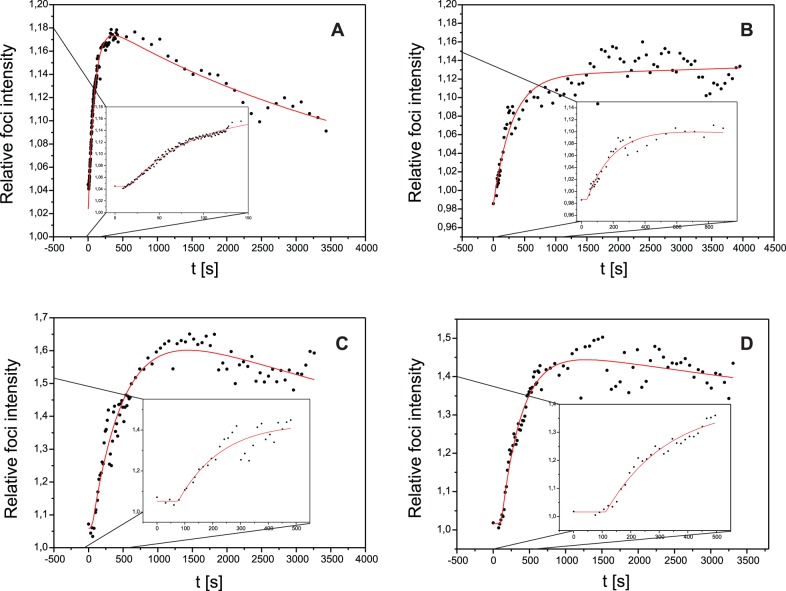
Foci intensity vs. time after irradiation. The relative foci intensity I_rel_  =  I_foci_/I_nucl_ of Mdc1 after irradiation with 5.2 Gy of 43 MeV carbon ions (A) and 4.8 Gy of 20 MeV protons (B) and 53BP1 after 7.6 Gy carbon (C) and 6.9 Gy proton irradiation (D) plotted for one cell and fitted with our model function (eq. 1). Irradiation took place on t  = 0. The insets show the protein accumulation after the irradiation with splayed time-axis.

Plots of this relative foci intensity I_rel_(t) of Mdc1 and 53BP1 in a single cell after irradiation with 43 MeV carbon ions or 20 MeV protons are shown exemplarily in fig. 2. It can clearly be recognized in the insets of fig. 2 that for both proteins there is a significant initial time lag T_0_ in which the intensity does not rise compared to the intensity before irradiation. This time lag period is followed by a steady intensity increase. At long time intervals an intensity decrease is observed. To quantify the data, a new kind of model function is fitted to I_rel_(t) that has not been used before to model kinetics of repair proteins:


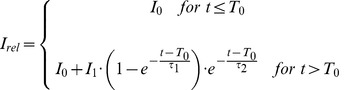
(1)

We introduce this kind of model function because it is well adapted to the measured data and can directly be interpreted in terms of the underlying kinetics of the molecules at the irradiation induced foci. The first part of this piecewise model function is characterized by a constant value of intensity ratio I_0_ =  I_foci_(0s)/I_nucl_(0s) during the time lag period of length T_0_, at which foci formation has not yet started. While I_0_ = 1 may be expected, it is not exactly obtained due to inhomogeneous Mdc1 distribution in the cell nucleus (e.g. at nucleoli etc.) before irradiation. At t  =  T_0_ foci formation starts, which can be described by the 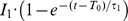 term in the second part of the piecewise function with τ_1_ representing the mean recruitment time. Concomitantly an intensity decrease takes place, e.g. due to successful repair, which is described by a mean decay time τ_2_ with τ_2_> τ_1_. I_1_ represents a kind of maximum intensity (above I_0_) the data would reach if there were no decline. Due to the relative measurement, I_1_ depends on the size of the region of interest, so that no useful or comparable information can be gathered from its value. The relevant parameters are T_0_, τ_1_ and τ_2_. These are determined for each cell separately. From these values error weighted mean values are calculated. The uncertainties of the mean values are evaluated from the fluctuations of the values obtained for each cell including the uncertainties of each fit. The error of T_0_ also contains the uncertainty of the exact time of irradiation of each individual cell.

### Mdc1 Kinetics

The following values were determined for Mdc1 in cell line U2OS pEGFP-Mdc1 after application of 5.2 Gy of 43 MeV carbon ions (see table 1; indicated are means and the standard errors of the means after analysis of 7 cells): Time lag T_0_ =  (17±2)s, mean recruitment time τ_1_ =  (98±11)s, and mean decay time τ_2_ =  (5300±1200)s. While the fluctuations of T_0_ and τ_1_ are small between different cells, τ_2_ deviates extensively from cell to cell. This is due to the limited time span of less than one τ_2_ of the kinetics investigation and possibly also to individual differences in repair kinetics, e.g. because cell cycle position of the cells and the individual damage structure vary from cell to cell. Since the emphasis of this investigation is on the recruitment kinetics, the mean decay time τ_2_ is not further discussed in this study, but it is used for correctly fitting the model function to all data presented in this work.

**Table 1:** Mdc1 kinetics.

Ion	Ions per point	Dose [Gy]	Number of cells	Time lag T_0_ [s]	Mean recruitment time τ_1_ [s]
43 MeV C	1	5.2	7	17±2	98±11
20 MeV H	128	4.8	22	73±16	1050±270
20 MeV H	320	12.1	10	80±11	520±150

Mdc1 kinetics after carbon and proton irradiation for U2OS pEGFP-Mdc1 clone F1. Indicated are means and the standard errors of the means of 7–22 cells per sample.

**Figure 3:**
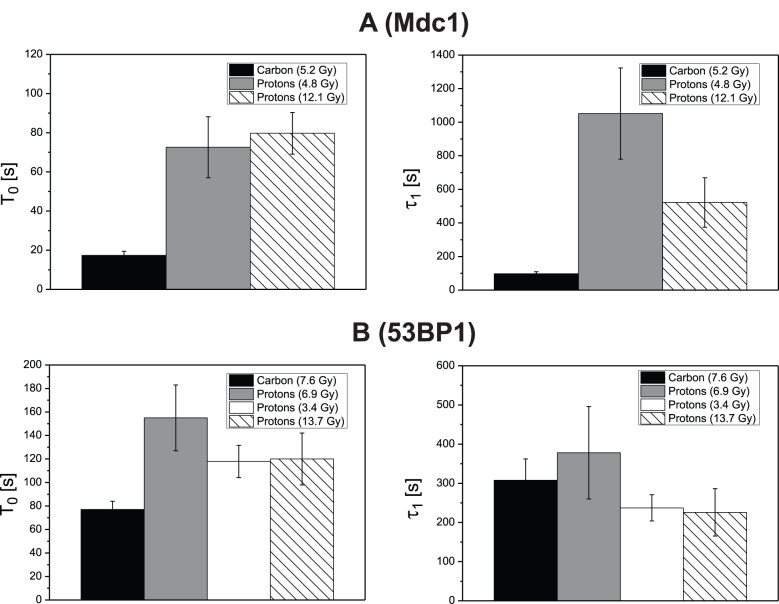
LET and dose dependence of recruitment kinetics. Weighted mean values and standard errors of the means of the kinetics parameters T_0_ and τ_1_ of the proteins Mdc1 (A) and 53BP1 (B) after irradiation with 43 MeV carbon ions and two or three different doses of 20 MeV protons.

The recruitment kinetics was also analyzed after irradiation with 20 MeV protons. The same irradiation pattern as with carbon ions was used, but in contrast to the carbon irradiation, where each point of the pattern was irradiated with one ion, 128 protons were applied to each point. This leads to a dose of 4.8 Gy that is comparable to the carbon dose. Fig. 3a and table 1 show the recruitment kinetics parameters T_0_ and τ_1_ after proton irradiation (grey) compared to the carbon irradiation (black). It is evident that the recruitment velocity after proton irradiation is significantly slower than after carbon irradiation, with T_0_ being about four times longer and τ_1_ about ten times longer. A similar influence of LET on both T_0_ and τ_1_ was observed in U2OS pMC16-Mdc1, demonstrating that the phenotype is not related to the deletion present in vector pEGFP-Mdc1 (see Material and Methods for description of the different cell lines).

To investigate if the Mdc1 recruitment kinetics depends on the absolute number of DSBs induced by the ions, the effect of irradiation with 320 protons per point was studied in U2OS pEGFP-Mdc1 (hatched in fig. 3a). This leads to a dose 2.3 times higher than used for carbon irradiation, so that according to the expected enhanced relative biological efficiency (RBE) for DSB production of 43 MeV carbon ions relative to 20 MeV protons the number of generated DSBs should be comparable in both irradiation modes, a single carbon ion or 320 protons per point [18]. T_0_ did not change significantly, but there is a tendency (p  = 0.097) for a reduction of the mean recruitment time τ_1_ by about 50% compared to the lower dose proton irradiation. In any case, τ_1_ was still a factor 5 longer than found for the carbon irradiation.

### 53BP1 Kinetics

Recruitment kinetics of 53BP1 was analyzed the same way as done for Mdc1. The results for Hela pMC16-53BP1-GFP clone #2 after irradiation with carbon ions and three different doses of protons are summarized in fig. 3b and table 2. After carbon irradiation, 53BP1 recruitment is slower than observed for Mdc1, with T_0_ about five times longer and τ_1_ about four times longer. The differences in T_0_ (p<0.0001) and τ_1_ (p  = 0.0029) are highly significant. But in contrast to Mdc1, no systematic dependence on ion or dose was observed for 53BP1 recruitment described by τ_1_ in the samples, which comprise a 7.6 Gy carbon irradiation and proton irradiations with a dose similar to the carbon dose (6.9 Gy), a lower dose of 3.4 Gy and a higher dose of 13.7 Gy. As a consequence and somewhat unexpectedly, 53BP1 shows a shorter mean recruitment time τ_1_ than Mdc1 after the low LET proton irradiation. It is important to note, however, that recruitment kinetics for both proteins were determined in different host cell lines and should thus not be compared directly.

**Table 2:** 53BP1 kinetics.

Ion	Ions per point	Dose [Gy]	Number of cells	Time lag T_0_ [s]	Mean recruitment time τ_1_ [s]
43 MeV C	1	7.6	17	77±7	310±60
20 MeV H	117	6.9	8	160±30	380±120
20 MeV H	58	3.4	12	118±14	240±40
20 MeV H	234	13.7	10	120±22	230±60
20 MeV H	Pooled		30	124±9	247±26

53BP1 kinetics (Hela pMC16-53BP1-GFP clone #2) after carbon and proton irradiation. Indicated are means and the standard errors of the means of 8–17 cells per data point. Because there was no significant difference between the various proton irradiations, pooled data are also shown in the last row.

With regard to T_0_, however, a significant difference (p<0.03) was observed between carbon irradiation and proton irradiation at a similar dose, suggesting that the duration of the lag phase is influenced by LET. Variation of proton dose, however, did not affect T_0._

### Rad52 Kinetics

**Figure 4:**
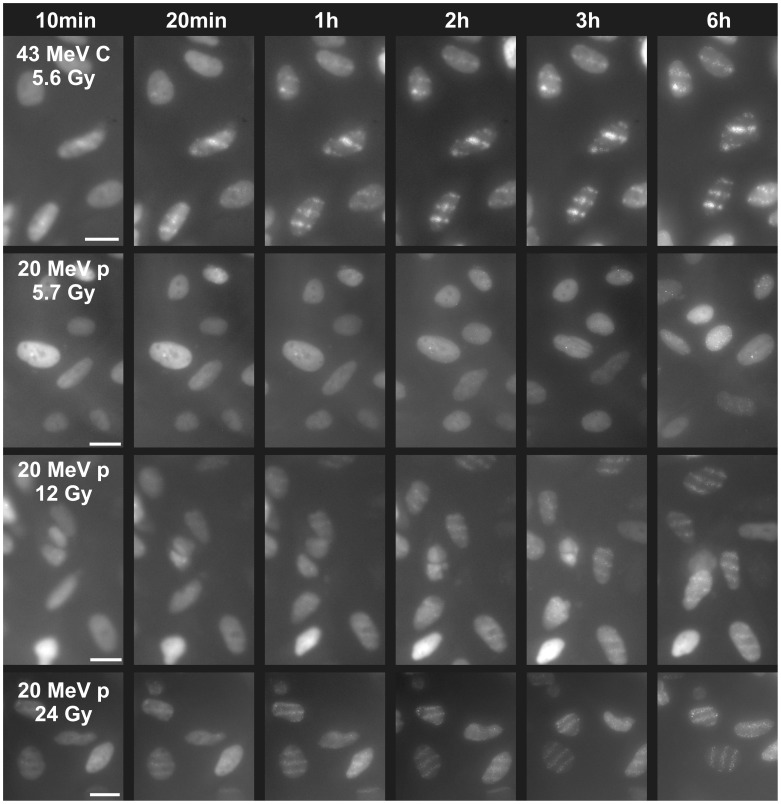
Dose and LET dependent kinetics of Rad52. 10–20 min after irradiation with 43 MeV carbon ions (5.6 Gy, one ion per point) foci become visible. When applying the similar dose by 117 20 MeV protons per point first very weakly developed foci become visible not before three hours have elapsed (upper right nucleus). Increasing the number of protons per point accelerates the kinetics; with 512 protons per point (i.e. 24 Gy) already ten minutes after irradiation foci can be seen, so that kinetics is similar to that of 5.6 Gy carbon irradiation. Scale bars 20 µm.

Investigation of the repair protein Rad52 shows slower recruitment kinetics than that of Mdc1 or 53BP1. Therefore and since the foci contrast was lower, the algorithm used for evaluation of Mdc1 and 53BP1 and described in Materials and Methods could not be applied to the data. However, gross information can be directly obtained from the images as presented in fig. 4. First foci could visually be recognized ten minutes after carbon irradiation with 5.6 Gy, and after 20 minutes they were clearly visible. For a similar dose applied with 20 MeV protons, however, it took about 3 hours until first foci could (barely) be seen. By increasing the number of protons per point and therefore the applied dose, the recruitment kinetics accelerated. When irradiating with 24 Gy by applying 512 protons per point, Rad52 recruitment appeared similar to that of 5.6 Gy 43 MeV carbon ions. Table 3 compares the time after which foci become visible by eye for all irradiation modalities that were performed.

**Table 3:** Rad52 kinetics.

Ion	Ions per point	Dose [Gy]	Time for foci forming (ca.)
43 MeV C	1	5.6	10 min
20 MeV H	117	5.7	3 h (barely)
20 MeV H	256	12	1 h
20 MeV H	512	24	<10 min

Rad52 kinetics after carbon and proton irradiation of cell pool U2OS pEGFP-Rad52. Indicated are the times after irradiation until foci formation is visible by eye in microscopic images.

### Comparison with UV Laser Irradiation

The results gathered in this work reflect cellular reactions on irradiation-induced DSBs as they occur e.g. due to cancer therapy or cosmic rays on earth or especially during space missions. The LET dependencies that we have found can only be discovered at a facility like the ion microprobe SNAKE which combines an ion microbeam of high and low LET ion species with a live-cell imaging environment. The often used UV laser microirradiation does not produce DSBs in such a predictable and “natural” way and is not able of revealing LET dependent effects. Nevertheless we set out to compare our results with data on UV laser irradiation found in literature, in order to test if the data in principle agree with each other.

Lukas et al. [30] studied Mdc1 recruitment kinetics for a time period of 700 seconds after irradiation, where no intensity decline is yet visible in their data. The authors did not recognize the time lag T_0_, maybe because of the lower number of time steps analyzed. They normalized their data so that the highest intensity equals one, pooled all evaluated cells and then fitted the pooled data using the simple model function


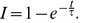
(2)

In order to be able to directly compare their data with ours, we also limited our time frame of the data gathered at SNAKE to 700 seconds after the irradiation, normalized, pooled and fitted them using the same simple function. The fitting results are presented in table 4. The comparison between laser and ion irradiation shows that the UV laser data rather correspond to the 43 MeV carbon irradiation (high LET irradiation) than to the proton irradiation (which can be considered as low LET).

**Table 4:** Mdc1 kinetics after ion irradiation compared with UV laser irradiation.

Irradiation with	Ions per point	Dose [Gy]	τ [s]
43 MeV C	1	5.2	148±3
20 MeV H	128	4.8	916±20
20 MeV H	320	12.1	557±17
UV laser	---	?	195.23±19.58

Laser data were taken from [18]. Recruitment times after carbon and proton irradiation data were determined using the fitting model proposed by Lukas et al. [18].

53BP1 recruitment kinetics was also analyzed the same way as Mdc1 by this group using UV laser irradiation [27]. Although the 53BP1 data showed a significant time lag T_0_, the authors did not interpret their data by adding a time lag period to the fitting but used a more complex fit function


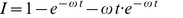
(3)

This “S”-shaped function has two drawbacks compared to a model introducing a time lag period: i) Eq. (3) describes a slightly rising intensity already right after irradiation, while we did not detect any intensity increase in this time period. ii) While our parameters have a concrete biological meaning (T_0_: time until foci forming starts, τ_1_: mean recruitment time of the protein accumulation), a biological interpretation cannot easily be drawn from the parameter ω used to describe the kinetics in [27]. The parameter ω in the first exponential term may also be interpreted as the inverse of the mean recruitment time, 1/τ_1_ but as ω also occurs in the second term it has also some influence on the S-shape of the function. As our data show a similar recruitment speed of 53BP1 for all used ion types and energies, we only modeled our 43 MeV carbon data according to eq. 3. The comparison is shown in table 5. Since the uncertainties are rather small, the differences in this kind of inverse recruitment time ω are significant, but still small. However, as already stated, the time lag period T_0_ was not recognized as such in [27] and there was no explanation given for choosing the model function eq. (3).

**Table 5:** 53BP1 kinetics after ion irradiation compared to UV laser irradiation.

Irradiation with	Dose [Gy]	ω [min^−1^]
43 MeV C	7.6	0.238±0.004
UV laser	?	0.35±0.01

### Discussion

The recruitment kinetics into irradiation-induced foci were analyzed for Mdc1, 53BP1 and Rad52 after low LET irradiation (20 MeV protons, 2.6 keV/µm) and high LET irradiation (43 MeV carbon ions, 370 keV/µm). The observed kinetics can be divided into three periods that can clearly be identified:

1. For each protein there is a time lag period T_0_ after irradiation in which no recruitment occurs. Apparently within this time period other processes, such as damage recognition, stimulation of the protein recruitment, or preparation of the binding sites, have to take place. Such a delay had not been considered in former studies of 53BP1 and Mdc1 [27], [30]. In our study, it can be clearly recognized due to the short time needed for irradiation and switching to microscopy and due to frequent acquisition of fluorescence images (every second). We note, however, that other proteins involved in the repair of DSBs or SSBs accumulate without exhibiting a detectable lag period [31], [32], [33].
2. After the lag period T_0_ the kinetics can be described as proportional to a single component kinetics of the kind 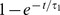
with a characteristic time constant τ_1_ which represents the mean time needed for the protein recruitment. Major factors influencing τ_1_ will be the time it takes for the protein to travel to the binding site, as well as the availability of binding sites.
3. This recruitment kinetics is superposed by depletion with an exponential decay rate 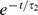, where the mean decay time τ_2_ describes the diminishment of the number of repair protein molecules, e.g. due to successful repair.

Our observations were limited in time, as especially the recruitment kinetics was the aim of the experiments. Therefore, the decay kinetics was not subject of this work, but taken into account for a correct fitting of all data. As τ_2_ is within the magnitude of hours, it is also possible to study such decay kinetics with conventional immunofluorescence methods [34].

In this work, we present for the first time systematically studied evidence that the recruitment kinetics of Mdc1 exhibits a strong dependence on LET as well as on the applied dose, regarding the time lag T_0_ and the mean recruitment time τ_1_. Concerning the dependence on LET, a similar tendency can be inferred from the study by Mosconi et al. [35], although these authors showed only preliminary data concerning the low LET irradiation. We show that the recruitment of Mdc1 takes place at an up to ten times higher speed after high LET irradiation (370 keV/µm) than after low LET irradiation (2.6 keV/µm) if the irradiation dose is similar. We tested the hypothesis that the faster recruitment kinetics after high LET irradiation results from the larger number of DSBs that are created by the high LET irradiation per unit dose. The number of DSBs per path length was determined by Monte Carlo simulations to be a factor f  = 2.2 larger for high LET irradiation of 250 keV/µm compared to our proton irradiation [18]. For our high LET irradiation with an LET of 370 keV/µm, the factor f is probably even a bit larger so that the proton dose used for this experiment was 2.3 times the dose used for the carbon irradiation in order to produce the same number of DSBs in both experiments. Although the recruitment kinetics of Mdc1 gets accelerated at the higher proton dose, it is still slower compared to that after high LET irradiation with lower dose. Thus, we attribute the faster recruitment kinetics of Mdc1 after high LET irradiation not only to a higher number of DSBs, but also to a higher spatial density of the DSBs (DSB clusters). This assumption is supported by the Mdc1 kinetics of a specimen which was irradiated by 43 MeV carbon ions not in a line pattern but in a 6 µm × 6 µm matrix shaped pattern. Thus the high DSB density in the ion tracks and thus the complexity of the damages are the same as of the other carbon irradiations, but the overall dose and therefore the number of DSBs induced is about a factor of three lower. In these cells a time lag T_0_ =  (10±3) s and a mean recruitment time τ_1_ =  (111±9) s is observed, which is not significantly different from the data gathered for the three times higher doses of linewise irradiation.

By comparing our data with the data obtained by Lukas et al. [30] after UV laser irradiation, we reveal that recruitment kinetics after UV laser irradiation resembles the kinetics after high LET ionizing irradiation rather than the kinetics after low LET irradiation. We conclude that UV laser irradiation may induce similarly large DSB density or complex lesions at DSB sites as it does high LET ionizing radiation. A similarity between damage induction by laser irradiation and high LET irradiation has also been noted by others [32], [36]. It should be noted that the similarity between UV laser and high LET ionizing irradiation is not necessarily true for foci disappearance in the course of repair.

While we provide qualitative data suggesting a similar LET and dose dependence for Rad52, we clearly show that 53BP1 behaves differently in that the mean recruitment time τ_1_ shows little dependence on LET and dose. A similar tendency can also be derived from the work by Mosconi et al. [35].

All three proteins tested in this work exhibit a shorter time lag T_0_ after irradiation with high LET radiation than after low LET irradiation. The main factors affecting the time lag T_0_ are damage recognition, stimulation of the protein recruitment, and preparation of the binding sites. It is still not clear how DSBs are initially sensed [37]: damage sensors like the Mre11-Rad50-Nbs1 (MRN)-complex may constantly diffuse along the DNA and bind to open ends once these are encountered [38]. Alternatively, a passive disturbance of the topological patterns of higher-order chromatin structure, as exemplified by break-induced relaxation of superhelical DNA loops, may trigger or facilitate DSB recognition by these end-binding proteins. In the latter case, it is conceivable that higher DSB density associated with high LET radiation may produce a stronger signal and thus accelerate damage recognition. Alteration of higher-order chromatin structure (e.g. by treatment with hypotonic media) may suffice to activate ataxia-telangiectasia mutated (ATM) [39]. ATM is the central player in the damage response cascade and, among other reactions, responsible for phosphorylation of H2AX and thus preparation of the binding site of Mdc1.

The main factors affecting τ_1_ include the mobility of the protein in question, the mean distance it has to travel to its binding sites, and the availability of the binding sites. How can these factors be affected by the damage density? Individual nuclear proteins are more or less freely diffusing in the nucleus and their mobility is affected by the molecular mass of the protein (or the complex it is part of) [40] and by its transient binding interactions. While Mdc1 appears to form a complex with the MRN complex also in undamaged cells [21] and chromatin-bound Mdc1 has also been observed in undamaged cells [41], it is difficult to conceive how Mdc1 binding to other proteins or chromatin remote from damage sites would be affected by damage density. Since the irradiation patterns applied in our study were comparable for high and low LET irradiations, on the scale of about 1 µm the positions of the damage sites in the cell nucleus and therefore the distances to be traversed by proteins randomly distributed in the nucleus are also comparable. We therefore favor a model in which the availability of Mdc1 binding sites is the main factor responsible for the observed LET and dose dependence. Damage density would positively affect the generation of binding sites if the proteins involved in generating them were rather loosely bound to the damaged sites, leading to frequent dissociation and binding to close-by damage sites.

According to current models, the initial steps before Mdc1 foci formation include detection of the DSB, presumably by the MRN-complex, recruitment of ATM to the break site and ATM-mediated phosphorylation of histone H2AX at serine139 to create γ-H2AX [42]. Mdc1 is recruited via its binding to phosphorylated C-terminal end of γ-H2AX [23], [24]. Signal amplification is obtained by Mdc1’s ability to bind further MRN complexes. If the damage density is high, ATM or other factors required for ATM recruitment could rapidly bind to another target site should they dissociate from the first binding site, thus eventually creating new binding sites for Mdc1. In this model, the limiting step for Mdc1 accumulation would be the availability of binding sites, which depends amongst others on the mean traveling time of upstream factors to the target, which in turn would be smaller at high density than in cases where damage sites are further apart from each other.

The qualitative evaluation of Rad52 recruitment also revealed a dependence on LET and dose for the Rad52 recruitment, since high LET radiation produced much faster Rad52 accumulation than the same dose of proton radiation. Only very high doses of protons (24 Gy) lead to a Rad52 kinetics that was similar to that obtained by about 6 Gy carbon irradiation. Considering the different ways of analysis, it should be noted that the time scale (about 10 min) for recruitment of Rad52 after high LET/high dose irradiation is not very much longer than for example the needed for recruitment of 53BP1, although the current view is that Rad52 function is coordinated with Rad51 [43], binding and accumulation of which depends on prior steps such as end processing to create single-stranded overlaps [44].

53BP1 differs from Mdc1 and Rad52 with regard to LET dependence of the recruitment kinetics, since the mean recruitment time τ_1_ of 53BP1 is independent of LET and dose at least within the dose range from 3.4 Gy to 13.7 Gy studied in this work. Another group reports in a recent study, that 53BP1 forms foci twice as fast after irradiation with 2 Gy of X-rays compared to 0.1 Gy [45].

**Figure 5:**
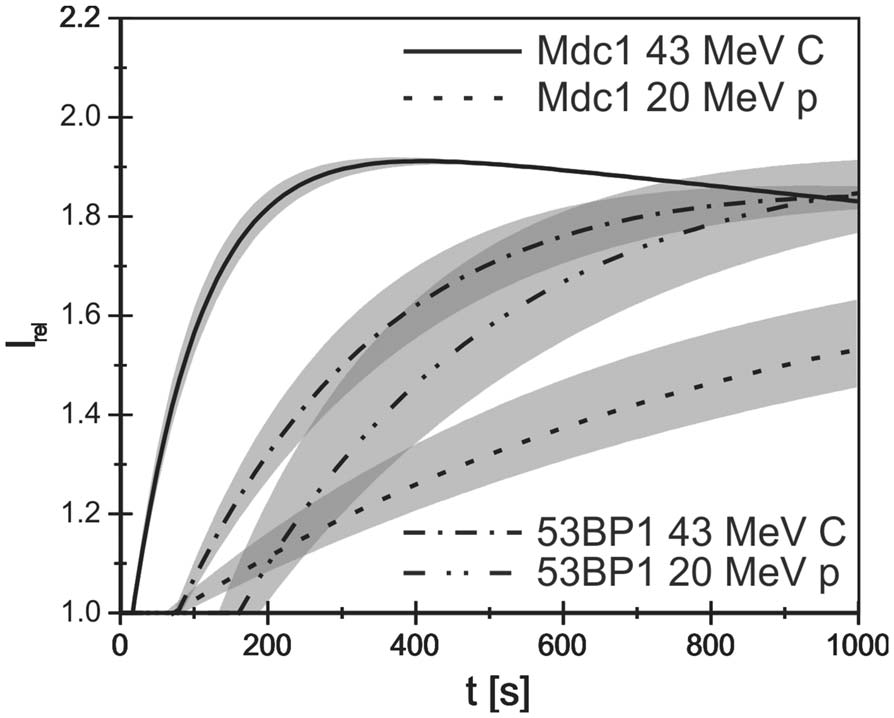
Comparison of the kinetics of Mdc1 and 53BP1 after carbon and proton irradiation. The shaded areas show the 1 σ error bands for the fit functions. A strong LET dependency of Mdc1 is visible. For 53BP1 only the time lag after which foci formation starts shifts from high LET to low LET. The models are based on data obtained after irradiation of U2OS pEGFP-Mdc1 clone F1 with 5.2 Gy carbon ions or 4.8 Gy protons, and Hela pMC16-53BP1-GFP clone #5 with 7.6 Gy carbon ions or 6.9 Gy protons, respectively.

For comparison between Mdc1 and 53BP1 kinetics under different irradiation conditions fig. 5 shows the fit function eq. (1) plotted using the mean values of T_0_, τ_1_ and τ_2_ as shown in tables 1 and 2 for Mdc1 and 53BP1 after carbon irradiation and proton irradiation with a similar dose. The data are normalized to I_0_ = 1 and I_1_ = 1. The shaded areas show the 1σ error bands for the fit functions. The figure demonstrates the strong dependence of Mdc 1 recruitment on the LET (carbon versus proton irradiation) as discussed. Comparing the plots of Mdc1 and 53BP1 after high LET (carbon) irradiation, it is evident that 53BP1 starts later with foci formation and exhibits a considerably slower recruitment speed, thus 53BP1 recruitment is slower than Mdc1 recruitment at all times after irradiation. The recruitment of 53BP1 after low LET (proton) irradiation is also shifted by a longer time lag T_0_ and thus starts later than that of Mdc1, but then the 53BP1 recruitment speed is about the same as after high LET irradiation, indicating that it is independent of damage density or complexity. As a consequence, under the experimental conditions used here, the recruitment functions for Mdc1 and 53BP1 cross each other and the recruitment of 53BP1 finishes earlier than that of Mdc1 after low LET irradiation. The reason for this behavior, which is in contradiction to generally accepted models claiming the 53BP1 recruitment depends on Mdc1, is not clear at present. It may simply be due to the different host cell lines used to measure the kinetics of the two proteins. On the other hand, the presence of a few Mdc1 molecules may already suffice to induce 53BP1 accumulation. In addition, Mdc1-independent initial binding and functions of 53BP1 have been demonstrated [46], [47], [48], [49]. In our hands, 53BP1 foci formation was found to be proficient also after knock-down of Mdc1 in HeLa cells, but not U2OS cells [50].

The uniformity of the mean recruitment time τ_1_ for 53BP1 might be explained by a limiting diffusion speed. Since the molecular weights of the 53BP1 molecules (∼220 kD) and the MDC1 molecules (∼230 kD) are similar, limited diffusion speed may be due to constitutive 53BP1 binding to undamaged chromatin [49]. Another possible explanation is that mean traveling distances are large for 53BP1 proteins because of limited protein abundance in the nucleus. Indeed in untransfected cells little residual 53BP1 protein not associated with the damage sites is found after irradiation [50]. Here, however, we used cells carrying 53BP1-GFP expressing vectors. Western blot analysis suggests that the total amount of 53BP1 protein (i.e. endogenous and exogeneous) amounts to about 140% of the 53BP1 amount present in untransfected cells (supplementary figure S2) and we did not find indications for limited 53BP1 supply.

In conclusion, our study demonstrates that LET-dependent variations in the kinetics of protein recruitment to DSB sites exist, the reasons of which have to be further analyzed. Given the high medical and biological importance of low LET radiation (X-rays, high energy electrons and protons), we propose that the DNA damage response and the interplay between the various proteins involved should not only be investigated under the convenient, but somewhat artificial conditions of UV laser irradiation, but also after low LET irradiation.

## Materials and Methods

### Cell lines

U2OS cells were stably transfected with pEGFP-Rad52 (to create a cell pool) or pEGFP-Mdc1 (to create clone F1). Both plasmids were generously provided by R. Kanaar, Rotterdam [51]. The Mdc1 ORF in pEGFP-Mdc1 inadvertently carries a deletion that leads to a loss of amino acids 1199–1239, i.e. one repeat of the 13 consecutive imperfect PST repeats (R. Kanaar, personal communication). The function of the PST domain has not yet been fully elucidated; it may be involved in DSB repair or mitotic functions [5]. Since the number of PST repeats in Mdc1 orthologs varies between organisms, the relative importance of the exact number of repeats present is not clear. In order to exclude effects due to this deletion, additional experiments were performed with a clone (#37) of U2OS cells stably transfected with bicistronic vector pMC16 Mdc1, a generous gift from W.G. Dirks, Braunschweig, which contains a wild-type Mdc1 ORF [35]. Total Mdc1 protein levels (endogenous plus GFP-tagged) for U2OS pEGFP Mdc1 and U2OS pMC16-Mdc1-GFP were 1.14 x and 1.79 x higher than the endogenous level present in untransfected U2OS cells (see supplementary figure S1 and Materials and Methods S1). Hela cells were stably transfected with pMC16-53BP1-GFP to generate clone #2. To generate the vector, the 53BP1 ORF was obtained by PCR from vector DKFZ p686B146Q2 and cloned into bicistronic pMC16 (a gift from W.G. Dirks, Braunschweig [52]). Plasmid sequence was verified by sequencing. Total 53BP1 protein levels in Hela pMC16-53BP1-GFP #2 is 1.37 x higher than the endogenous level present in untransfected HeLa cells (see supplementary figure S2).

Twelve until 72 hours before irradiation the cells were seeded into specially developed cell containers [10], where they grow on a 170 µm thick plastic scintillator BC-418 (for carbon irradiation), or a 170 µm thick cover glass (for proton irradiation), each pre-treated with CellTAK (BD Biosciences) to enhance adhesion. Cells were cultivated in HEPES-buffered, phenol red-free medium supplemented with 0.25 mM Trolox at 37° and 5% CO_2_. At the time of irradiation, the cells are in exponential growth phase.

### Irradiation

The cells were irradiated at the target station of the Munich ion microprobe SNAKE. During the irradiation and the subsequent observation procedure the cells were covered by cell culture medium and the temperature was always kept at 37°C. Single 55 MeV carbon ions – counted by a photomultiplier tube using the scintillation light from the plastic scintillator to which the cells adhered – were applied in a line-shaped point pattern in a similar way as described [14]. As the ions have to traverse a stack of 7.5 µm Kapton foil, 5 µm Mylar foil, and about 20 µm cell culture medium, they lose about 12 MeV, leading to an energy of about 43 MeV and therefore to an LET of about 370 keV/µm at the cell position. To vary the average dose per cell nucleus between 3.7 Gy and 12 Gy, the x-distance between two points within a line was varied for different experiments between 1 and 2 µm, the distance between two lines between 5 and 8 µm. The pointing accuracy of the pattern is about 700 nm full width at half maximum [10]. Irradiation of a field of about 150×120 µm^2^ takes about three seconds with this set-up. Microscopy was started within one second after the irradiation has finished. Irradiations with 20 MeV protons (LET  = 2.6 keV/µm), which do not significantly slow down when traversing the two foils and the culture medium, were performed applying the same line pattern, but the number of protons applied per point was varied between 58 and 512 in order to adjust the average dose between 3.4 Gy and 24 Gy. Depending on the number of protons applied per point, irradiation takes up to 15 seconds. As 20 MeV protons are not stopped in the scintillator, the cells are cultivated on a standard cover slip and the protons are counted by a scintillator-photomultiplier unit mounted to the objective revolver [14] to perform experiments with protons. Therefore the time needed for switching from irradiation to microscopy increases to 30–60 seconds. Since the protons arrive in a beam spot of similar size as that of the carbon ions (∼700 nm full width at half maximum), they are practically homogeneously distributed along each line of irradiation when using a point to point distance of 1 µm.

### Microscopy

Microscopy was performed by a Zeiss Axiovert 200 M inverse epifluorescence microscope, which had been tilted by 90° and mounted to the horizontal beamline as a part of the SNAKE live-cell imaging setup [10]. A “Zeiss Plan-Apochromat 40x/0.95 Korr Ph3” objective that does not require any immersion media (e.g. oil) was used for easy handling at the tilted microscope. Its numerical aperture of 0.95 ensures sufficient image brightness and an optical resolution of about 320 nm according to the Rayleigh criterion. It provides an optical correction for the 5% refraction index mismatch between the plastic scintillator on which the cells are cultivated for carbon ion irradiation and a standard glass coverslip, which is normally used as a cell substrate for high resolution microscopy. Illumination was performed by a commercially available LED light source for fluorescence microscopy (Zeiss Colibri), which reduces photobleaching and phototoxicity effects significantly as compared to a mercury arc lamp, since no UV light is emitted. Microscopic images were gathered by a Zeiss AxioCam MRm CCD camera. By using the Zeiss AxioVision software, time series with several segments were taken. When observing proteins with fast recruitment kinetics like Mdc1, for the first two minutes after irradiation every second an image was taken, and later on the time intervals between two acquisitions were extended.

### Quantitative Analysis

In order to perform quantitative analysis of the time-dependent recruitment of the repair factors, image analysis of the GFP fluorescence was performed for each cell nucleus in the microscopic field separately because the cells move and rotate in plane independently. The cell nucleus in question was cut off a time series of images and processed by the open source image analysis software, imageJ. In a first step, the images were corrected for lateral movements of the cell nucleus as well as for their rotations in x-y. For lateral corrections of each image the center of the cell nucleus was determined. Then each of the images was re-positioned so that the centers are aligned. For rotation correction the brightness profile along a circle within the nucleus was determined. Starting with the first image in the time series, the next images were rotated until the deviation of each profile from the initial one was minimized. These automatic movement corrections can be checked and corrected manually, if necessary.

In a second step the region of interest (ROI), which represents the entity of all foci sites for all images of the time series, is determined. For that purpose the images of the time series are subtracted pairwise (e.g. image 50– image 1, image 51– image 2 etc.). The result of each subtraction reveals those pixels the fluorescence intensity of which has changed with time. All those pixels of all subtractions are summed up to the ROI. This approach is illustrated in figure 1. To avoid adding noise to the ROI, pixels are only added if the result of the subtraction for this pixel exceeds a pre-defined threshold for at least two subtractions. Once the region of interest was determined, for all images of the time series the ROI is used as a kind of mask, that is put onto the images, and the mean intensity per pixel within the ROI is evaluated, which represents the foci intensity I_foci_(t) at a certain time t. That means I_foci_(t) is the mean intensity of all pixels of the image taken at the timepoint t, that have the same coordinates as the white pixels of the ROI shown in fig. 1. To correct for photobleaching effects, the intensity I_foci_(t) has to be normalized to the mean intensity per pixel I_nucl_(t) of the whole cell nucleus for each image, resulting in I_rel_(t)  =  I_foci_(t)/I_nucl_(t).

Time synchronization between the SNAKE control software and the image acquisition software AxioVision ensures that the time t can be declared relative to the timepoint of irradiation (which is t  = 0 in all graphs presented in this work). The time needed for irradiation (which took about three seconds as mentioned above) was recorded for each sample and from that time an uncertainty of t was determined, which is included in the declared uncertainties of T_0_ via Gaussian error propagation.

## Supporting Information

Figure S1 **Quantification of the MDC1 and MDC1-GFP expression level in U2OS pEGFP-MDC1 F1 and U2OS pMC16-MDC1-GFP #37**. Whole cell extracts of the indicated cell lines were immunoblotted and the MDC1 and MDC1-GFP expression was quantified by Western-Blot analysis. (A) MDC1 and MDC1-GFP expression was determined by immunoblottin analysis using an antibody probe specific for MDC1. Immunoblotting with Tubulin-α was done to show equal loading. (B) MDC1 and MDC1-GFP expression levels of the indicated cell lines. The immunoblotting signal of MDC1 was normalized to the Tubulin-α signal as determined with the Bio-1D software (Vilber Luormat).
(TIF)

Figure S2 **Quantification of the 53BP1 and 53BP1-GFP expression level in Hela pMC16-MDC1-GFP #37**. Whole cell extracts of the indicated cell lines were immunoblotted and the 53BP1 and 53BP1-GFP expression was quantified by Western-Blot analysis. (A) 53BP1 and 53BP1-GFP expression was determined by immunoblotting analysis using an antibody probe specific for 53BP1. Immunoblotting with SMC1 was done to show equal loading. (B) 53BP1 and 53BP1-GFP expression levels of the indicated cell lines. The immunoblotting signal of 53BP1 was normalized to the SMC1 signal as determined with the Bio-1D software (Vilber Luormat).
(TIF)

Materials and Methods S1 **Immunoblotting and quantitative Western analysis.**
(DOC)
