# Supplementary figures and images for: Recruitment Kinetics of DNA Repair Proteins Mdc1 and Rad52 but Not 53BP1 Depend on Damage Complexity
Source: PLoS One. 2012 Jul 30;7(7):e41943. doi: 10.1371/journal.pone.0041943 (PMC3408406; doi:10.1371/journal.pone.0041943)

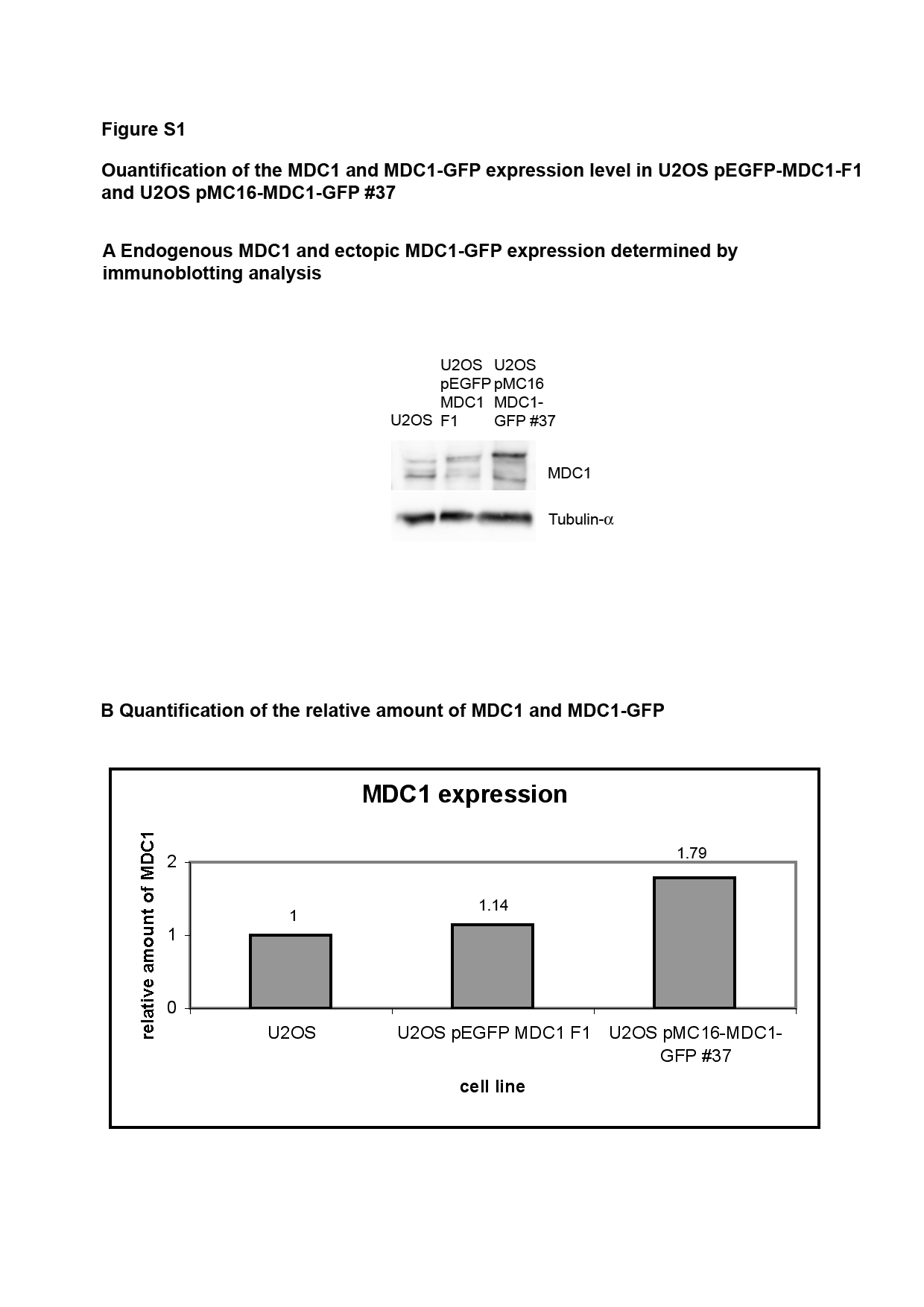

Supplement: Figure S1 — Quantification of the MDC1 and MDC1-GFP expression level in U2OS pEGFP-MDC1 F1 and U2OS pMC16-MDC1-GFP #37. Whole cell extracts of the indicated cell lines were immunoblotted and the MDC1 and MDC1-GFP expression was quantified by Western-Blot analysis. (A) MDC1 and MDC1-GFP expression was determined by immunoblottin analysis using an antibody probe specific for MDC1. Immunoblotting with Tubulin-α was done to show equal loading. (B) MDC1 and MDC1-GFP expression levels of the indicated cell lines. The immunoblotting signal of MDC1 was normalized to the Tubulin-α signal as determined with the Bio-1D software (Vilber Luormat). (TIF) [file pone.0041943.s001.tif]

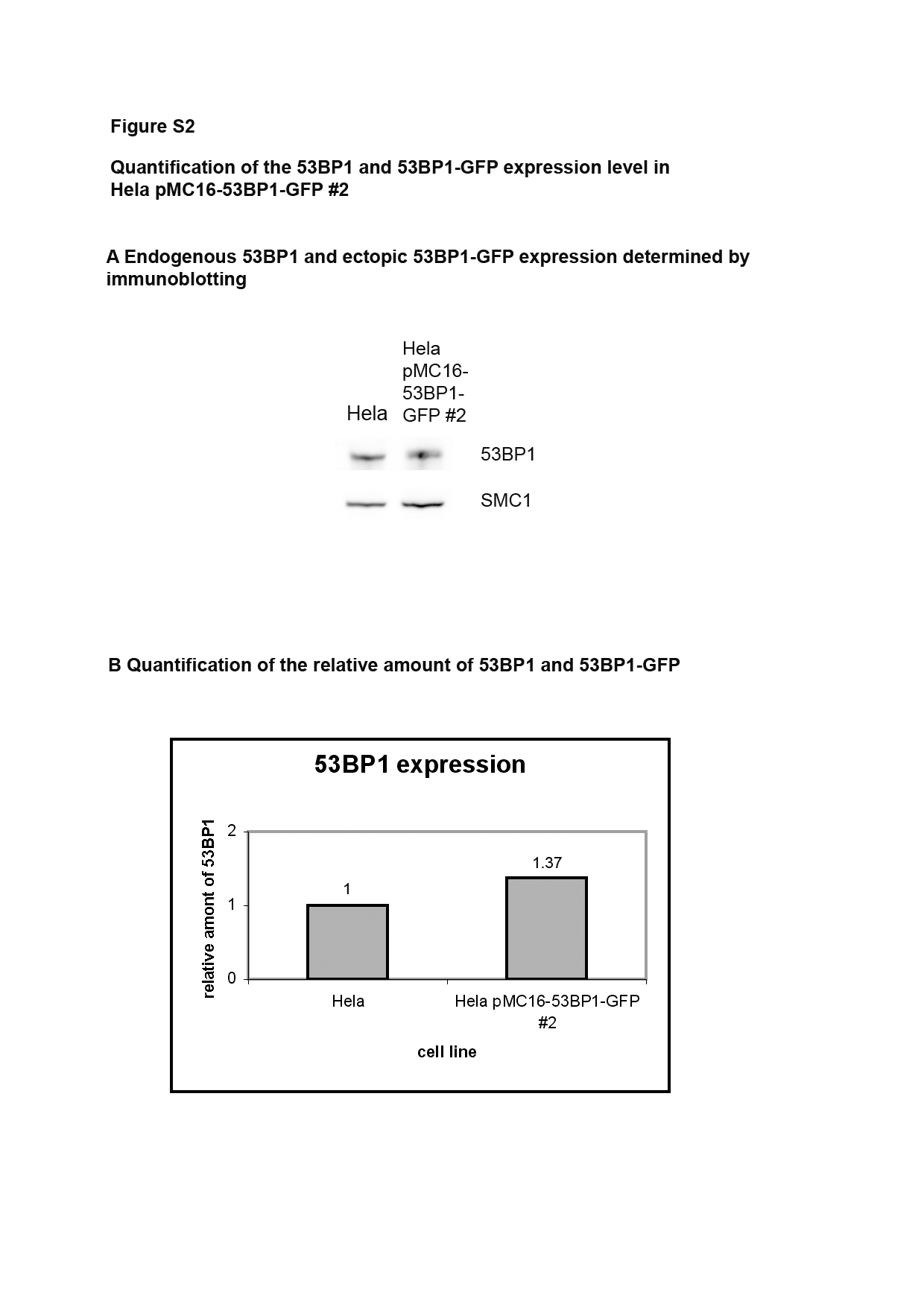

Supplement: Figure S2 — Quantification of the 53BP1 and 53BP1-GFP expression level in Hela pMC16-MDC1-GFP #37. Whole cell extracts of the indicated cell lines were immunoblotted and the 53BP1 and 53BP1-GFP expression was quantified by Western-Blot analysis. (A) 53BP1 and 53BP1-GFP expression was determined by immunoblotting analysis using an antibody probe specific for 53BP1. Immunoblotting with SMC1 was done to show equal loading. (B) 53BP1 and 53BP1-GFP expression levels of the indicated cell lines. The immunoblotting signal of 53BP1 was normalized to the SMC1 signal as determined with the Bio-1D software (Vilber Luormat). (TIF) [file pone.0041943.s002.tif]
