# Supplementary material for: Recruitment Kinetics of DNA Repair Proteins Mdc1 and Rad52 but Not 53BP1 Depend on Damage Complexity
Source: PLoS One. 2012 Jul 30;7(7):e41943. doi: 10.1371/journal.pone.0041943 (PMC3408406; doi:10.1371/journal.pone.0041943)
Supplement: Materials and Methods S1 — Immunoblotting and quantitative Western analysis. (DOC) [file pone.0041943.s003.doc]

### **Materials and Methods S1**

*Immunoblotting and quantitative Western analysis*

Cells were grown until semiconfluency and proteins were extracted with RIPA-buffer (150 mM NaCl, 1 % NP-40, 10 mM MDOC, 0,1% SDS 50 mM Tris pH 8.0) on ice. After denaturation for 10 min at 104 °C, protein concentration was determined with the BCA^TM^ Protein Kit (Thermo Scientific) according the manufactures instructions. 5 µg protein were separated with Leammli loading dye on 3-8% Tris-Acetate NuPAGE-gels (Invitrogen). After immunoblotting, membranes were cut and blocked with 5% bovine serum albumin (BSA) in PBS supplemented with 0,1% Tween-20. After blocking the apropiate membrane pieces were incubated with the primary antibodies rabbit anti-MDC1 (Bethyl, A300-051), rabbit anti-53BP1 (Novus Biologicals, NB100-305), rabbit anti-SMC1 (Abcam, ab21583) and mouse anti-Tubulin-α (Abcam, ab7291) respectively, in 5% BSA in PBS supplemented wit 0,1% Tween-20 before detection with appropiate secondary antibodies anti-mouse-HRP and anti-rabbit-HRP (Santa Cruz, sc2004 and sc2005 respectively). Blots were detected with Amersham ECL Advance (GE Healthcare) and TMA-6 (Lumigen) respectively. Chemoluninescence images were aquired with a CHEMISMART documentation System (peqlab, Vilber Luormat) and the Chemi-Capt software. Quantitative analysis was realized with the Bio-1D software (Vilber Luormat). MDC1 and 53BP1 signals were normalized with respect to the tubulin-α or SMC1 signals.
